# L-glutamine protects against enterohemorrhagic *Escherichia coli* infection by inhibiting bacterial virulence and enhancing host defense concurrently

**DOI:** 10.1128/spectrum.00975-23

**Published:** 2023-10-10

**Authors:** Fang Fang, Yunxin Xue, Xuefang Xu, Dingli Fang, Weijia Liu, Ying Zhong, Jinping Han, Yunhe Li, Qian Tao, Rong Lu, Cong Ma, Arvind Kumar, Dai Wang

**Affiliations:** 1 Department of Laboratory Medicine, Xiamen Key Laboratory of Perinatal-Neonatal Infection, Women and Children's Hospital, State Key Laboratory of Vaccines for Infectious Diseases, Xiang An Biomedical Laboratory, School of Public Health and School of Medicine, Xiamen University, Xiamen, Fujian Province, China; 2 State Key Laboratory of Infectious Disease Prevention and Control and National Institute for Communicable Diseases Control and Prevention, Chinese Center for Disease Control and Prevention, Changping, Beijing, China; 3 Department of Pathology, Women and Children's Hospital, State Key Laboratory of Molecular Vaccinology and Molecular Diagnostics, National Innovation Platform for Industry-Education Integration in Vaccine Research, Xiamen University, Xiamen, Fujian Province, China; 4 Department of Nephrology, Lishan Hospital, Anshan Central Hospital, Anshan, Liaoning Province, China; 5 BiomEdit, Fishers, Indiana, USA; Cinvestav-IPN, Mexico City, Mexico

**Keywords:** enterohemorrhagic *Escherichia coli*, L-glutamine, nitrogen metabolism, type 3 secretion system, antivirulence, host defense

## Abstract

Enterohemorrhagic *Escherichia coli* (EHEC) can colonize the gastrointestinal tract and cause bloody diarrhea in children. Previous studies showed that its pathogenesis could be mediated by metabolites from both the host and microbiota. L-Glutamine (Gln) was found to be depleted in intestinal tissues as the main energy source according to previous studies. Hence, we aimed to determine the effects of Gln on EHEC infection and its underlying mode of action. In this study, a Gln-limited signal was found to activate the type 3 secretion system (T3SS), which is crucial for EHEC infection *via* perturbation of central metabolism. By shifting the phosphorylation of NtrC, a key regulator in bacterial nitrogen metabolism, Gln stimulates *ler* transcripts in a σ^S^-PchA-dependent manner. Our *in vivo* experiments further demonstrated that Gln supplementation can reduce EHEC colonization in the gastrointestinal tract by repressing T3SS. Moreover, Gln could further attenuate bacterial infection by boosting host defense, which might be dependent on multiple pathways. Besides, our experiments demonstrated that Gln did not induce Shiga-like toxin (Stx) production or cause impairment of gut flora. In conclusion, our study presented evidence that Gln could act against EHEC infection by reducing bacterial virulence and strengthening host defense. Therefore, Gln serves as a promising therapeutic agent for EHEC infection.

**IMPORTANCE:**

The type 3 secretion system (T3SS) was obtained in many Gram-negative bacterial pathogens, and it is crucial for their pathogenesis. Environmental signals were found to be involved in the expression regulation of T3SS, which was vital for successful bacterial infection in the host. Here, we discovered that L-glutamine (Gln), the most abundant amino acid in the human body, could repress enterohemorrhagic *Escherichia coli* (EHEC) T3SS expression *via* nitrogen metabolism and therefore had potential as an antivirulence agent. Our *in vitro* and *in vivo* evidence demonstrated that Gln could decline EHEC infection by attenuating bacterial virulence and enhancing host defense simultaneously. We repurpose Gln as a potential treatment for EHEC infection accordingly.

## INTRODUCTION

As an important food-borne zoonotic pathogen, enterohemorrhagic *Escherichia coli* (EHEC) can cause infection, resulting in bloody diarrhea or even life-threatening hemolytic uremic syndrome (HUS) in humans (1, 2). EHEC not only produces Shiga-like toxin (Stx), which results in major tissue damage in patients clinically (3, 4) but also possesses a type 3 secretion system (T3SS), which is a protein complex structure forming a hollow conduit extending through bacterial double membranes to the host membrane (5). *Via* this T3SS nanomachine, EHEC could inject bacterial effectors into host cells directly, which was essential for EHEC intestinal colonization and caused subsequent pathological changes in epithelial cells, namely, attaching and effacing (AE) lesions (6).

The pathogenicity island responsible for AE lesions was identified and named as the locus of enterocyte effacement (LEE) in EHEC, enteropathogenic *Escherichia coli* (EPEC), and *Citrobacter rodentium* (CR) earlier, which contains five operons (LEE1-5) encoding >50 proteins required for a functional T3SS, including translocator proteins EspADB, basal structure proteins, chaperone proteins, and T3SS regulator proteins—Ler/GrlAR (6 – 9). Previous studies have proved that LEE gene expression was regulated by environmental cues, such as temperature, pH (10), oxygen availability (11), and other stimuli.

The mammalian gastrointestinal tract is a stimulus-rich environment with small molecules/metabolites from microbial communities and the host (12, 13). Bacterial pathogens can sense specific signals through various mechanisms and regulate their virulence expression, thus colonizing the intestinal tract and forming ecological niches rapidly (13). L-glutamine (Gln) is the most abundant free amino acid in the human body and plays an essential role in epithelial cell renewal, intestinal paracellular permeability, redox status, and immune functions under stress or pathological conditions (14 – 20). Hence, Gln has been widely applied as a nutrition supplement in post-surgery recovery, cancer treatments, and noninfectious diarrhea clinically (21, 22). As Gln is consumed as the main energy source for intestinal cells, the concentration of Gln was found to be much lower in intestinal tissues (23), the main sites for EHEC colonization, compared to that found in other organs/tissues. Therefore, in this study, we explored the impact of Gln on EHEC infection and its underlying mechanisms.

## MATERIALS AND METHODS

### Bacterial strains and cell lines

The bacterial strains, plasmids, and oligonucleotides used in the study are described in Tables S1 and S2. The isogenic mutants were generated by a CRISPR (clustered regularly interspaced short palindromic repeat)/Lambda RED-based method and verified by PCR amplification and sequencing. The open reading frame of the gene and its promoter region were inserted into pWSK29 to construct the complementary plasmid, and then the plasmid was transformed into the corresponding strain. Bacteria were cultured at 37℃ (200 rpm) in Luria broth (LB) or MEM-HEPES (MEM) as required. Cell lines were cultured in DMEM at 37°C with 5% CO_2_ unless stated. Antibiotics were added when needed: chloramphenicol (Chl) 12.5 µg mL^−1^, kanamycin (Kan) 25 µg mL^−1^, spectinomycin (Spec) 100 µg mL^−1^, and ampicillin (Amp) 50 µg mL^−1^.

### RNA-seq library preparation and analysis

An RNA-seq approach was used to compare EHEC strain ZAP193 gene expression in the presence and absence of different nitrogen supplementations. Total RNA extracted from biological samples was used to perform strand-specific RNA-seq experiments. Sequencing was done at Novogene and BGI. Briefly, mRNA was enriched by removing rRNA from total RNA using a specialized kit (Novogene) or streptavidin magnetic beads (BGI), followed by treatment with fragmentation buffer to break mRNA into short fragments. First-strand cDNA was synthesized using random hexamer primers and mRNA as templates, followed by synthesis of the corresponding double-stranded cDNAs. The purified double-stranded cDNAs were first end-repaired, A-tailed, and ligated to the sequencing linker; fragments (150–200 bp) were amplified and purified to obtain the final library. All samples were sequenced on an Illumina HiSeq platform. HTSeq v0.6.1 (Novogene) or Bowtie v2.2.5/RESM v1.2.8 (BGI) was used to count the read numbers mapped to each gene. Data analysis was performed using DESeq software, and reads were compared with the genome of *E. coli* O157:H7 strain Sakai (National Center for Biotechnology Information NC_002128.1). For DEGseq without biological replicates, the *P* values were adjusted using the Benjamini-Hochberg method (statistical significance: corrected *P* value < 0.05).

### Quantitative real-time PCR

RNA was extracted from three biological replicates using TriPure RNA Isolation Reagent (Roche). According to the manufacturer’s protocols, cDNA was then synthesized using the 5× All-in-One RT MasterMix kit (Abcam). Subsequent quantitative real-time PCR (qRT-PCR) was performed in a CFX96 touch real-time PCR system instrument (Bio-Rad) with SYBR green PCR master mix (Takara Bio). The relative abundance of mRNA transcripts was normalized to the 16S rRNA gene and analyzed using the threshold cycle (∆∆*C_T_*) method (24).

### Secreted protein analyses

Bacteria were cultured overnight in LB (37°C, 200 rpm) and re-inoculated into fresh MEM (Sigma) at 37°C (200 rpm) to an OD_600_ of 0.8 unless stated. Culture supernatants were obtained at 4,000 × *g* for 30 min and filtered (0.45 µm). BSA (bovine serum albumin) was added to supernatant samples as a loading control when needed. Then, protein pellets were obtained by centrifugation at 4,000 × *g* for 30 min (4°C) after TCA (trichloroacetic acid) precipitation overnight (10%, 4°C). Protein samples were resuspended in 90 µL 1.5 M Tris (pH 8.8) buffer and separated by 12% SDS-PAGE, followed by Coomassie blue staining or immunoblotting (the EspA polyclonal antibody and EspD monoclonal antibody were kindly provided by Prof. David L. Gally at Edinburgh University) with standard methods described previously (25). All SDS-PAGE/immunoblotting experiments have been carried out at least in triplicate (statistics were performed using a *t* test or ordinary one-way ANOVA multiple comparisons), and only representative results are shown.

### Analysis of fluorescence levels

GFP-fused reporter plasmids were transformed into EHEC and cultured at a T3 permissive condition. The fluorescence produced by bacteria was detected by measuring 150-µL aliquots of culture with a fluorescent plate reader (FLOUstar Omega, BMG). Each experiment was carried out at least six times independently, which all showed similar tendencies (Fig. S2), and only representative results are shown.

### Fluorescence staining assays

Cells grew overnight to 70–80% confluence (37°C, 5% CO_2_) on coverslips in high-glucose (4.5 g/L) DMEM with 1% penicillin-streptomycin and 10% fetal bovine serum (FBS). Two hours before infection, DMEM was discarded and replaced with fresh MEM without antibiotics/FBS. Then, cells were infected with log-phase bacterial cultures using standard protocols with a multiplicity of infection (MOI) of 10. One hour later, the cells were washed with per-warmed medium to remove unattached bacteria and further incubated for another 3 h with fresh medium. After that, cells on coverslips were washed, fixed, and permeabilized. Then, bacteria were detected with the O157 monoclonal antiserum (gift from Prof. Ruifu Yang) and secondary antibody (Alexa Fluor 488). Cells were treated with FITC (fluorescein isothiocyanate) -labeled phalloidin and DAPI (4',6-diamidino-2-phenylindole) to visualize F-actin and cell nuclei, respectively. The samples were mounted on slides and imaged with an Olympus FV1200 confocal microscope (Olympus, Japan). Bacteria/cell numbers were then counted under a fluorescence microscope for statistical analyses (% cell with pedestals = cells with pedestal/total cells). At least six fields of each slide were imaged and counted for statistical analysis (>60 Caco-2 cells or >123 HeLa cells found in each field).

### Animal experiments

Animal experiments refer to the experimental methods of Mohawk et al. (26). The protocol was approved by the Animal Ethics Committee at Xiamen University (Permission No. XMULAC20220280). Four-week-old female BALB/c mice (C57BL/10ScNJ mice were used for mortality analysis) were randomly divided into different groups according to body weight. Mice were given either sterilized water (Gln limited [GL]) or sterilized water containing 150 mM L-glutamine (Gln rich [GR]), respectively. One week later, mice were intragastrically infected with EHEC (10^9^ CFU) or CR (10^9^ CFU). The number of bacteria excreted in mouse feces was monitored for consecutive days after infection. At different time points, the small intestine near the cecum, cecum, rectum, and blood sample were collected from mice, and the intestinal contents were gently scraped away. Subsequently, the intestine was washed with PBS three times, weighed, and homogenized in NaCl (0.1 g/mL). After that, the homogenates were diluted and plated on LB agar with nalidixic acid resistance (50 µg mL^-1^). For mortality analysis, the weight and health of the mice were monitored/recorded daily. Commercial ELISA (enzyme linked immunosorbent assay) kits were used for measuring the concentrations of KC (RK00038, ABclonal), MIP-2 (RK04208, ABclonal), and IL-17 (RK00039, ABclonal) in serum according to the manufacturer’s instruction.

### Analysis of the composition of intestinal microbiota

Murine stool samples were collected 1 week after different Gln administrations. Genomic DNA was extracted using HiPure Soil DNA Kits (Magen, Guangzhou, China). The V3-V4 hypervariable region of 16S rRNA genes was amplified by PCR using 341F/806R. PCR products were extracted and purified from 2% agarose gels and quantified using a QuantiFluor fluorometer. According to standard protocols, samples were pooled and cleaned in equimolar amounts and paired-end sequenced (PE250) on an Illumina platform. Raw reads were obtained and filtered by FASTP. Clean tags were aligned to the reference database to perform reference-based chimera checking by the UCHIME algorithm. Nonbacterial tags and all chimeric tags were removed. Operational taxonomic unit (OTU) clustering was performed with the UPARSE pipeline, using abundance-based greedy clustering.

## RESULTS

### T3SS was down-regulated by Gln *via* LEE repression in EHEC

**Fig 1:**
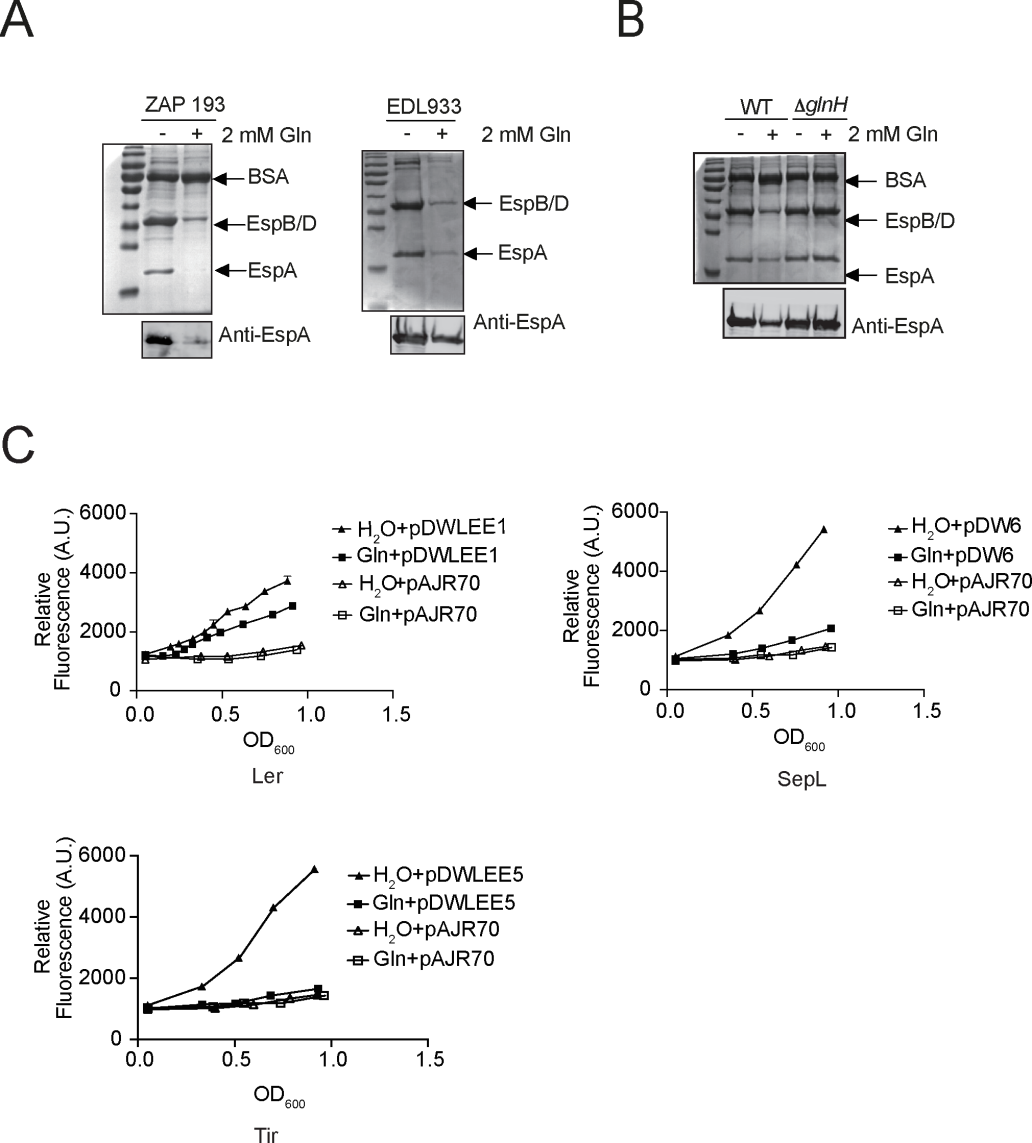
Gln inhibits T3SS *via* LEE in EHEC. (**A**) T3 secreted proteins of different EHEC strains were visualized using Coomassie blue staining, and EspA was detected by immunoblotting using an EspA polyclonal antibody following culture with or without 2 mM Gln supplementation. (**B**) Gln transportation is necessary for Gln-mediated T3SS repression. T3 secreted proteins of EHEC wild-type (WT) and Δ*glnH* strains were visualized using Coomassie blue staining, and EspA was detected by immunoblotting with an EspA polyclonal antibody following culture with or without 2 mM Gln supplementation. (**C**) Expression levels of Ler (pDWLEE1), SepL (pDW6), Tir (pDWLEE5), and empty control (pAJR70) were monitored with GFP fusion plasmids in EHEC strain ZAP193 when cultured with or without 2 mM Gln. (The results of one representative experiment out of at least six that were performed with similar results are shown.) Abbreviations: EHEC, enterohemorrhagic *E. coli*; Gln, glutamine; LEE, locus of enterocyte effacement; T3SS, type 3 secretion system.

As one of the most abundant amino acids found in human bodies, lower Gln concentrations (0.2~0.5 mM) were found in intestinal tissues rather than in other organs/tissues (23, 27, 28). In this study, experiments were carried out to examine whether Gln limitation could serve as a niche signal for EHEC colonization. EHEC was cultured with different Gln supplementation, and secreted proteins were then separated by SDS-PAGE and visualized by Coomassie blue staining. Our results showed that Gln-limited conditions might activate T3SS-associated protein secretion in EHEC strain ZAP193 in a dose-dependent manner, as demonstrated in Fig. 1A; Fig. S1 and S3A. This phenotype was further examined by immunoblotting using an EspA/D antibody, as evidenced in Fig. 1A. In order to rule out the possibility of a strain-specific effect, it was further tested with another EHEC strain EDL933, which was isolated in a previous outbreak, and our results confirmed that Gln could inhibit T3SS in EHEC strain EDL933 as well (Fig. 1A; Fig. S3A). Therefore, our results here revealed that Gln could repress T3SS in EHEC. Furthermore, LEE expression was evaluated in EHEC under those growth conditions using GFP reporter plasmids. Our data showed that LEE1, 4, and 5 expressions were all reduced by the addition of 2 mM Gln in the culture media (Fig. 1C; Fig. S2), suggesting that Gln repressed EHEC T3SS by modulating LEE expression.

### Gln transportation is necessary for Gln-mediated T3SS repression

As reported previously, Gln is transported into *E. coli* through ABC transporters encoded by *glnH/P*/*Q,* and GlnH is a periplasmic binding protein that affects Gln transportation (29). To investigate whether Gln could regulate the expression of virulence genes in EHEC extracellularly, we constructed a *glnH* mutant that did not uptake Gln in the medium and tested whether Gln could still reduce T3-related protein secretion in this *glnH* mutant. Our results showed that Gln was no longer able to inhibit protein secretion in the Δ*glnH* strain, while deleting *glnH* did not cause any impairment of T3SS in EHEC when cultured in the medium without Gln supplementation (Fig. 1B; Fig. S3B). In conclusion, our experiments clearly suggested that Gln reduces the expression of EHEC virulence genes intracellularly and that its transportation is crucial for this regulation.

### Transcriptomic analysis of EHEC cultured with different nitrogen supplementations

**Fig 2:**
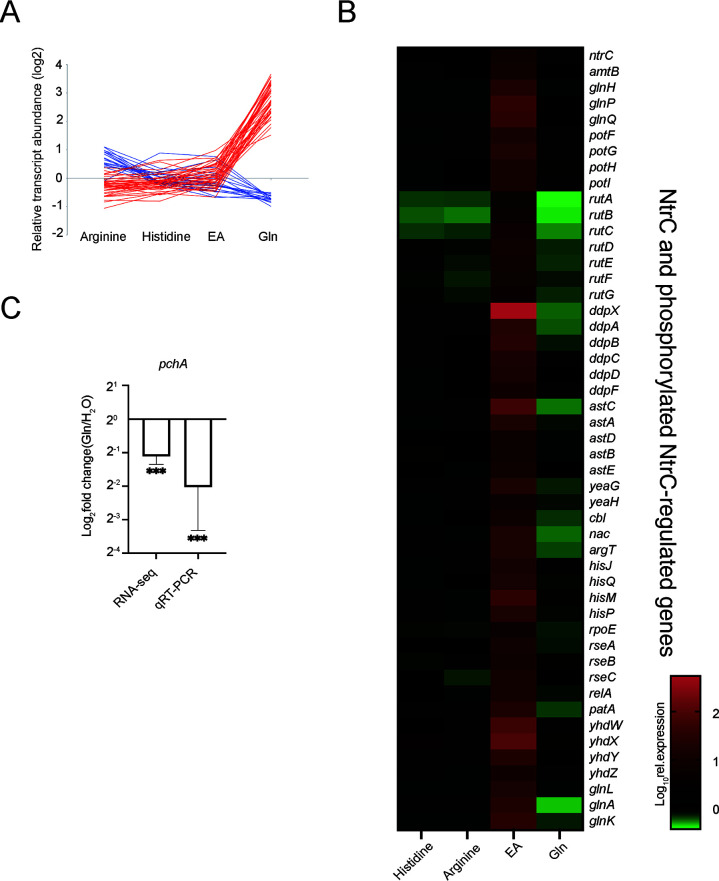
Transcriptomic analysis of EHEC exposed to different nitrogen sources. (**A**) RNA-seq analysis of the expression of LEE and flagella genes of EHEC cultured in the presence of various nitrogen sources, respectively. Only genes with significant changes (>1.42-fold, *P* < 0.05) after Gln addition are shown in the figure. T3SS-associated genes (blue); flagellar-associated genes (red). (**B**) RNA sequence analysis of the expression of NtrC and phosphorylated NtrC-regulated genes of EHEC cultured in the presence of various nitrogen sources, respectively. (**C**) RNA sequence analysis of the expression of *pchA* of EHEC cultured with or without 2 mM Gln supplementation. To verify the RNA-seq analysis, transcript levels of *pchA* (*n* = 3) were evaluated by quantitative real-time PCR (qRT-PCR). Statistics were performed using an unpaired *t* test. Data are mean ± standard deviation (SD). **P < 0.01, ***P < 0.001. Abbreviations: EA, ethanolamine; EHEC, enterohemorrhagic *E. coli*; Gln, glutamine; LEE, locus of enterocyte effacement; T3SS, type 3 secretion system.

Nitrogen was a key nutrient for microbes, and previous studies tried to explore its role in EHEC infection (30). However, previous studies were inconclusive, as different nitrogen sources had diverse effects on EHEC colonization. In order to further understand the mechanism of LEE repression caused by Gln, our study examined expression patterns of EHEC strain ZAP193 cultured in media with different nitrogen supplementations, respectively, such as Gln, histidine, arginine, and ethanolamine (EA), using RNA sequencing. Surprisingly, LEE genes were repressed in EHEC by Gln rather than by histidine, arginine, or EA (Fig. 2A). Apart from LEE repression, our data revealed that the expression of flagellum- and nitrogen metabolism-relevant genes was also found significantly affected in EHEC due to Gln supplementation (Fig. 2A and B; Table S3).

### Transcriptional regulation of *ler* is mediated by phosphorylated NtrC *via* σ^S^/PchA

**Fig 3:**
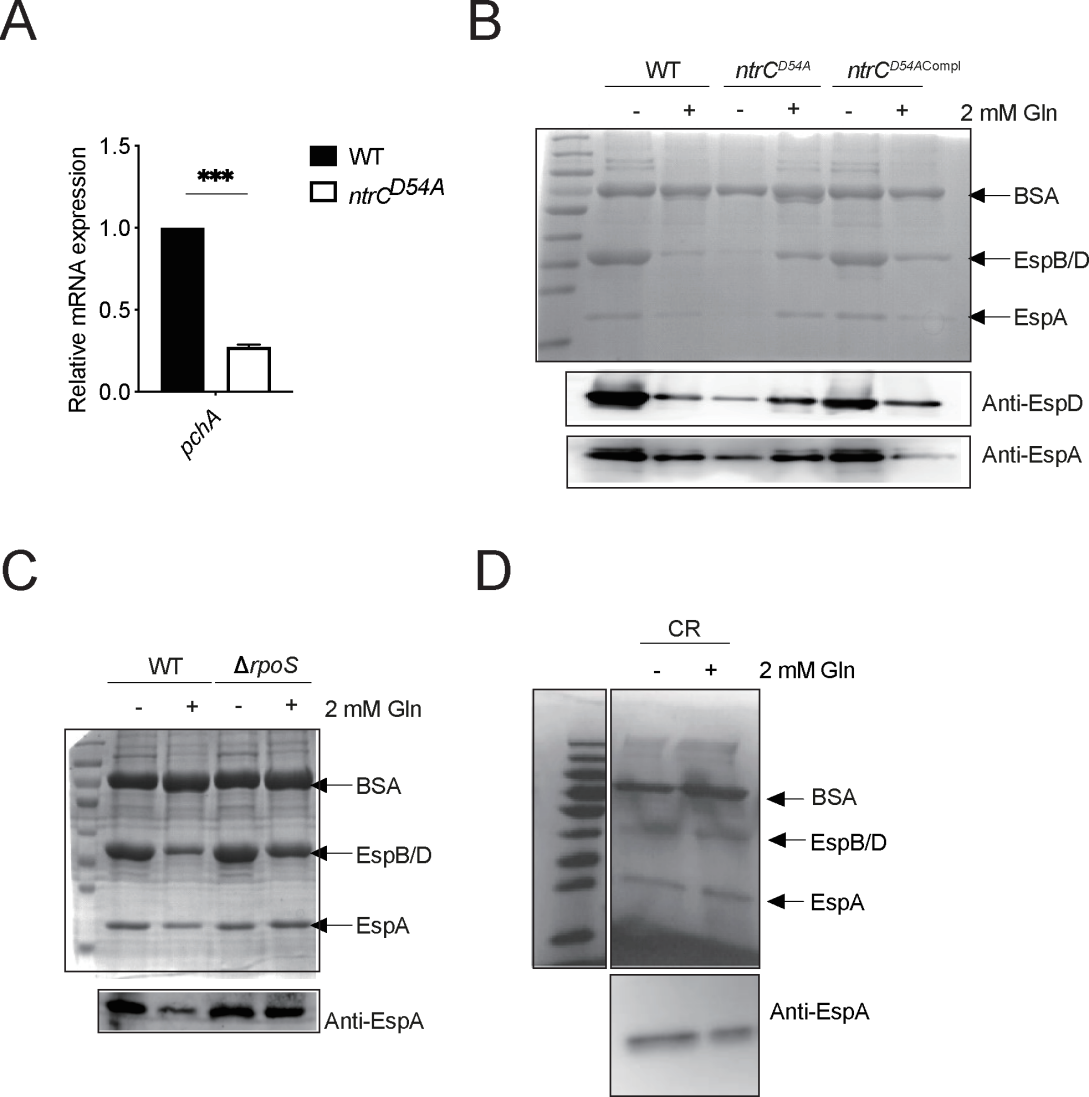
Transcriptional regulation of *ler* is mediated by phosphorylated NtrC *via* σ^S^ and PchA. (**A**) Transcript levels of *pchA* were evaluated by quantitative real-time PCR in EHEC wild-type (WT) and isogenic *ntrC^D54A^* strains (*n* = 3). Statistics were performed using an unpaired *t* test. Data are mean ± standard deviation (SD). ***P < 0.001. (**B**) T3 secreted proteins of EHEC WT, *ntrC^D54A^*, and *ntrC^D54A^* ^Compl^ strains were visualized using Coomassie blue staining, and EspA/D was detected by immunoblotting following culture with or without 2 mM Gln supplementation. (**C**) The secreted protein profiles of EHEC WT and ∆*rpoS* strains were visualized using Coomassie blue staining, and EspA was detected by immunoblotting using an EspA polyclonal antibody following culture with or without 2 mM Gln supplementation. (**D**) Gln does not repress T3SS in *C. rodentium* (CR). The secreted protein profiles of CR were visualized using Coomassie blue staining, and EspA was detected by immunoblotting using an EspA polyclonal antibody following culture with or without 2 mM Gln supplementation. Abbreviations: EHEC, enterohemorrhagic *E. coli*; Gln, glutamine; T3SS, type 3 secretion system.

As shown above, Gln could not only affect T3SS/flagellum expression but also affect gene expression patterns related to central metabolism in EHEC. Therefore, we speculated that Gln might inhibit LEE expression by interfering with bacterial metabolism. Nitrogen metabolism is essential for the growth and development of microbes, and nitrogen limitation controls the expression of genes of the nitrogen-regulated (Ntr) response in bacteria. Triggered by low nitrogen conditions, NtrC is a key regulator that is accumulated and partially phosphorylated, activating the transcription of *glnA*/*K* and other genes involved in the Ntr response (31, 32). It was also observed that *glnA*/*K* transcripts were reduced significantly in the Gln-supplemented sample, while *ntrC* transcripts were unaltered in our transcriptomic results (Fig. 2B and the supplemental material). Thus, we sought to investigate the role of NtrC phosphorylation in LEE expression regulation in response to Gln, and an EHEC strain containing an NtrC unphosphorylation mutation (NtrC^D54A^) was constructed consequently for this aim. Our results demonstrated that the secretion of translocons was impaired by the NtrC^D54A^ mutation (Fig. 3B; Fig. S3C). Our investigation with this NtrC^D54A^ mutant further confirmed that Gln no longer exerted its inhibitory effect on bacterial T3SS under the condition of NtrC signaling pathway blockade (Fig. 3B; Fig. S3C). These results showed that LEE expression was repressed by Gln in EHEC, possibly by changing the phosphorylation level of NtrC.

As NtrC has been implicated in the regulation of σ^S^ activity in EHEC before, it was likely that Gln-mediated LEE repression occurs by down-regulating *ler* transcripts in a σ^S^-PchA-dependent manner (33). It was also evident in our RNA-seq and RT-PCR results that *pchA*/*ler* transcripts were down-regulated in EHEC by 2 mM Gln addition (Fig. 2C). Moreover, our data also demonstrated that the NtrC^D54A^ mutation significantly reduced the expression of *pchA* (Fig. 3A) and that the loss of σ^S^ hindered Gln-mediated T3SS repression in EHEC (Fig. 3C; Fig. S3D). Consistent with previous reports, our study also confirmed that deleting *pchA* lessened secreted proteins in EHEC (Fig. S3E). Thus, Gln-repressed LEE expression in EHEC was likely mediated by NtrC phosphorylation, which modulated *ler* expression *via* σ^S^ and PchA.

### Gln does not repress T3SS in CR

Bioinformatics screenings suggested earlier that PchA/PerC regulation was missing in CR, and therefore, it was not expected that Gln-mediated repression of T3SS could be observed in this pathogen. To examine our hypothesis, experiments were carried out to clarify the effect of Gln on T3SS in CR*.* Bacteria were cultured in the media with or without additional 2 mM Gln, and samples of bacterial secreted proteins were harvested when OD_600_ reached 0.8. Samples of bacterial secreted proteins were then separated by SDS-PAGE and visualized by Coomassie blue staining. As expected, T3-associated protein secretion was not attenuated in CR in response to Gln. Also, this observation was further confirmed by EspA immunoblotting and by quantification of *ler* transcripts (Fig. 3D; Fig. S4).

**Fig 4:**
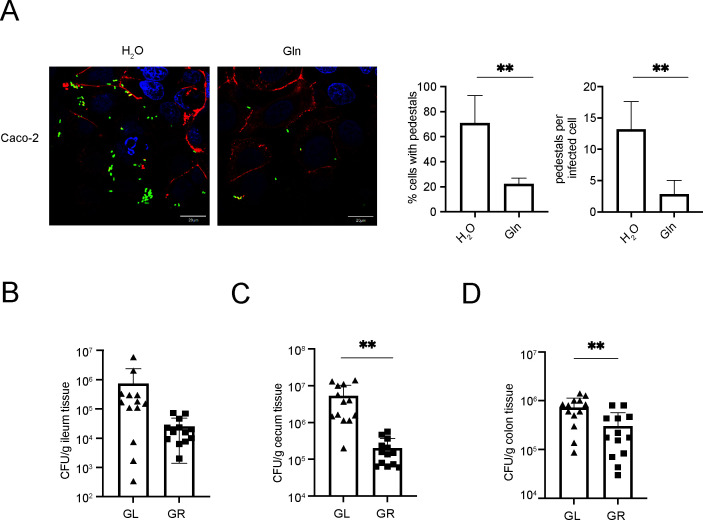
Gln supplementation decreases EHEC colonization in cells and *in vivo.* (**A**) Attaching and effacing (AE) lesion formation on Caco-2 cells by EHEC in the absence or presence of 2 mM Gln. EHEC forms AE lesions on epithelial cells, whose hallmark is actin accumulation underneath the bacterial cell forming a pedestal-like structure. EHEC were probed with an O157 antibody and Alexa Fluor 488-conjugated secondary antibody (green). Cells were stained with FITC-labeled phalloidin (actin, colored in red) and DAPI (DNA, colored in blue) and observed with fluorescence microscopy (×100), Scale bars, 20 µm. Percentage of cells infected with EHEC pedestals and quantification of EHEC pedestals per infected cell. Statistics were performed using an unpaired *t* test. Data are mean ± standard deviation (SD). **P < 0.001. (**B to D**) Adherence capacity of EHEC in the ileum, cecum, and colon of mice fed with a Gln-limited diet (GL) or a Gln-rich diet (GR). Each symbol represents an individual mouse. Statistics were performed using an unpaired *t* test. Data are mean ± standard deviation (SD). **P < 0.01. Abbreviations: EHEC, enterohemorrhagic *Escherichia coli*; Gln, glutamine.

### Gln attenuates T3SS-dependent EHEC interactions with host cells

EHEC can utilize T3SS for its interaction with host cells and form “pedestal” structures on epithelial cells, which featured polymerized actin cytoskeleton accumulation underneath attached bacteria. As Gln was shown to repress T3SS in EHEC, it was then tested for its impact on EHEC-epithelial cell interactions. As demonstrated in our study, in the presence of 2 mM Gln, bacterial attachment was found impaired on epithelial cells (Caco-2 and HeLa cells) relative to the results observed in the control experiments, which were cultured in the medium without Gln supplementation (Fig. 4A; Fig. S5B). Moreover, pedestal formation on epithelial cells by EHEC was also attenuated when 2 mM Gln was added (Fig. 4A; Fig. S5B). Therefore, our results clearly suggested that Gln could inhibit T3SS-dependent EHEC interactions with host cells.

**Fig 5:**
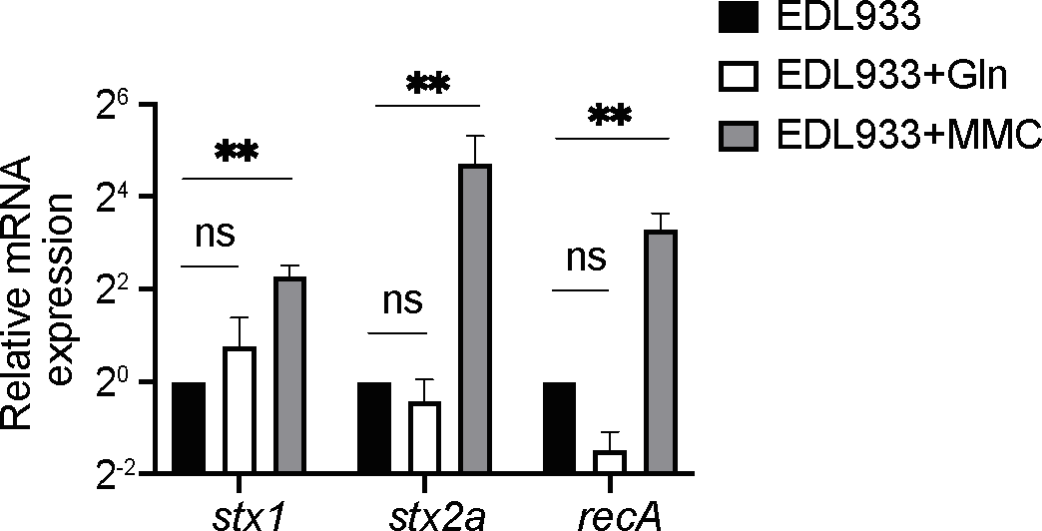
Gln does not induce *stx* expression. Quantitative real-time PCR evaluating the transcription of *stx1*, *stx2*, and *recA* of EHEC cultured in the absence or presence of 2 mM Gln. MMC was used as a positive control, *n* = 3. Statistics were performed using an unpaired *t* test. Data are mean ± standard deviation (SD). **P < 0.01. Abbreviations: EHEC, enterohemorrhagic *Escherichia coli*; Gln, glutamine.

### Gln supplementation decreases EHEC colonization *in vivo*

As demonstrated above in our study, Gln could attenuate T3SS-dependent EHEC interactions with host cells. However, it was still unknown whether Gln could inhibit EHEC colonization *in vivo*. To explore this, a murine infection model with intact commensal flora was adapted in our study. Two groups of mice (*n* = 13) were provided with water with Gln (GR) or without Gln (GL) for 1 week before infection and then orally infected with EHEC (1 × 10^9^ CFU). It was found that the amount of EHEC colonized in the GR mouse intestine was much less than the amount in the GL mouse intestine at 6 h postinfection. In particular, it was clearly demonstrated in our study that Gln could reduce bacterial loads in the cecum/rectum, which were previously reported as major colonization sites for EHEC in mice (Fig. 4B through D). It was speculated that the reduction in bacterial colonization was mainly due to T3SS repression caused by Gln administration, while the interference of the host immune response might be minor in such a limited time of infection. Thus, our data support that dietary Gln could reduce early bacterial colonization by weakening T3SS and present a potential treatment for EHEC/EPEC infection.

### Gln does not induce *stx* expression

Antimicrobial treatment might cause DNA damage and subsequently stimulate the SOS response, activating the expression of bacteriophage-encoded Stx in EHEC (34). Consequently, as a potential solution for EHEC infection, Gln was tested for its effects on *stx* expression in this study. Our RNA-seq data did not reveal the differential expression of SOS-regulated genes (35) in *E. coli* O157 strain ZAP193 (not containing the *stx* gene) triggered by Gln (supplemental material). To validate this, qRT-PCR was performed to examine the expression of *recA,* encoding a key regulator for the SOS response and *stx* in another *E. coli* O157 strain EDL933 in response to Gln. Total RNA was extracted from bacterial pellets, and gene expression was then measured by qRT-PCR. Mitomycin C was used as an inducer of their expression (36) and triggered log-fold activation compared with the uninduced control. In contrast, the data show that Gln does not stimulate either *recA* or *stx* transcription in EHEC after 6 h of induction (Fig. 5). Our results imply that the presence of Gln does not trigger the SOS response or *stx* transcription, strengthening the potential of this amino acid to be used for treating EHEC infections.

### Gut microbiome composition was unaltered following Gln supplementation

**Fig 6:**
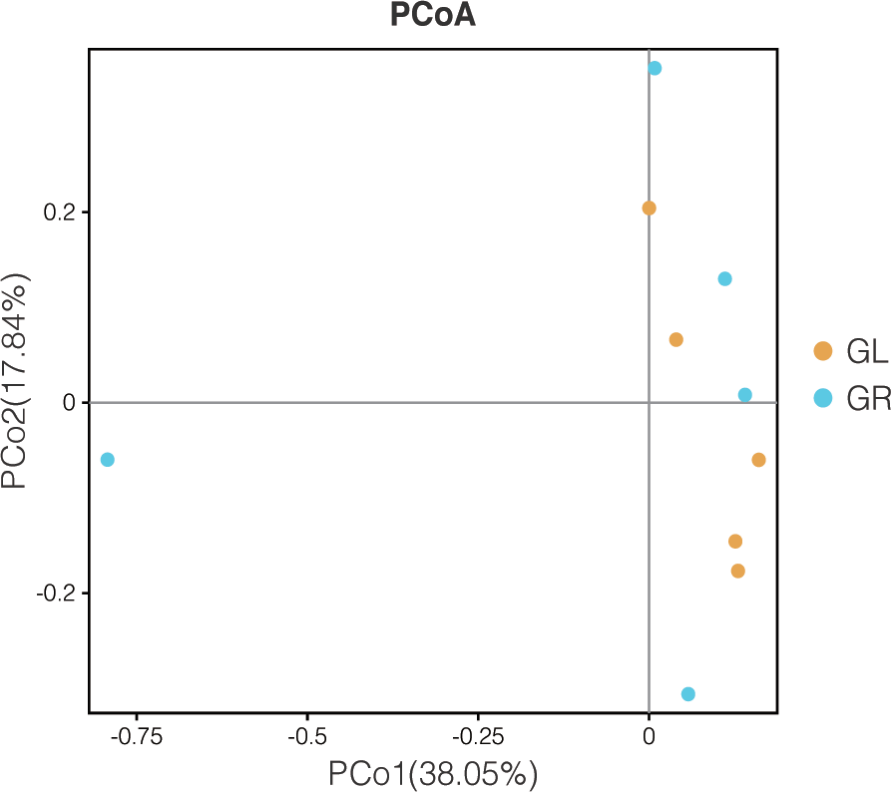
Gut microbiome composition was unaltered following glutamine (Gln) supplementation. Mice were fed for 7 d with a Gln-limited diet (GL) or a Gln-rich diet (GR). After sequencing, the principal coordinate analysis (PCoA) plot of microbial species abundance using the different Gln diets as a grouping variable, based on the Bray-Curtis distances, was established. Statistical significance was assessed *via* PerMANOVA and ANOSIM. *n* = 5 mice per group.

Beyond disease development, the gut microbiome has been proved critical for the most fundamental host physiological phenotypes. The treatment of antimicrobial or other agents often causes gut microbiome impairment or even worse dysbiosis. In order to further explore the effect of Gln as a treatment for EHEC, an *in vivo* experiment was carried out to verify its impact on gut flora. Two groups of mice were kept on normal chow supplemented with different drinking water (GL or GR), and mouse feces were collected after 1 week for gut microbiome identification using 16S rDNA sequencing. The effects of dietary Gln supplementation on gut flora are presented in Table 1; Fig. 6. As presented here, our results showed that GR diets did not affect the abundance or diversity of intestinal microbes, according to the Chao and ACE metrics, Shannon diversity index, and Simpson diversity index (Table 1). The beta diversity distance matrix was visualized using principal coordinate analysis (PCoA), which showed no structural differences in the gut bacterial community between GR mice and GL mice (Fig. 6). In conclusion, dietary Gln supplementation does not impair gut flora, as shown in this study.

**TABLE 1:** Effect of Gln on Alpha diversity of intestinal flora

	GL	GR
RICHNESS ESTIMATORS		
Chao	1229.093	955.74242
Ace	1281.9084	933.76885
DIVERSIITY ESTIMATORS		
Shannon	5.145586	4.357709
Simpson	0.934524	0.858679

### Gln protects against Stx-producing CR infection *in vivo*

**Fig 7:**
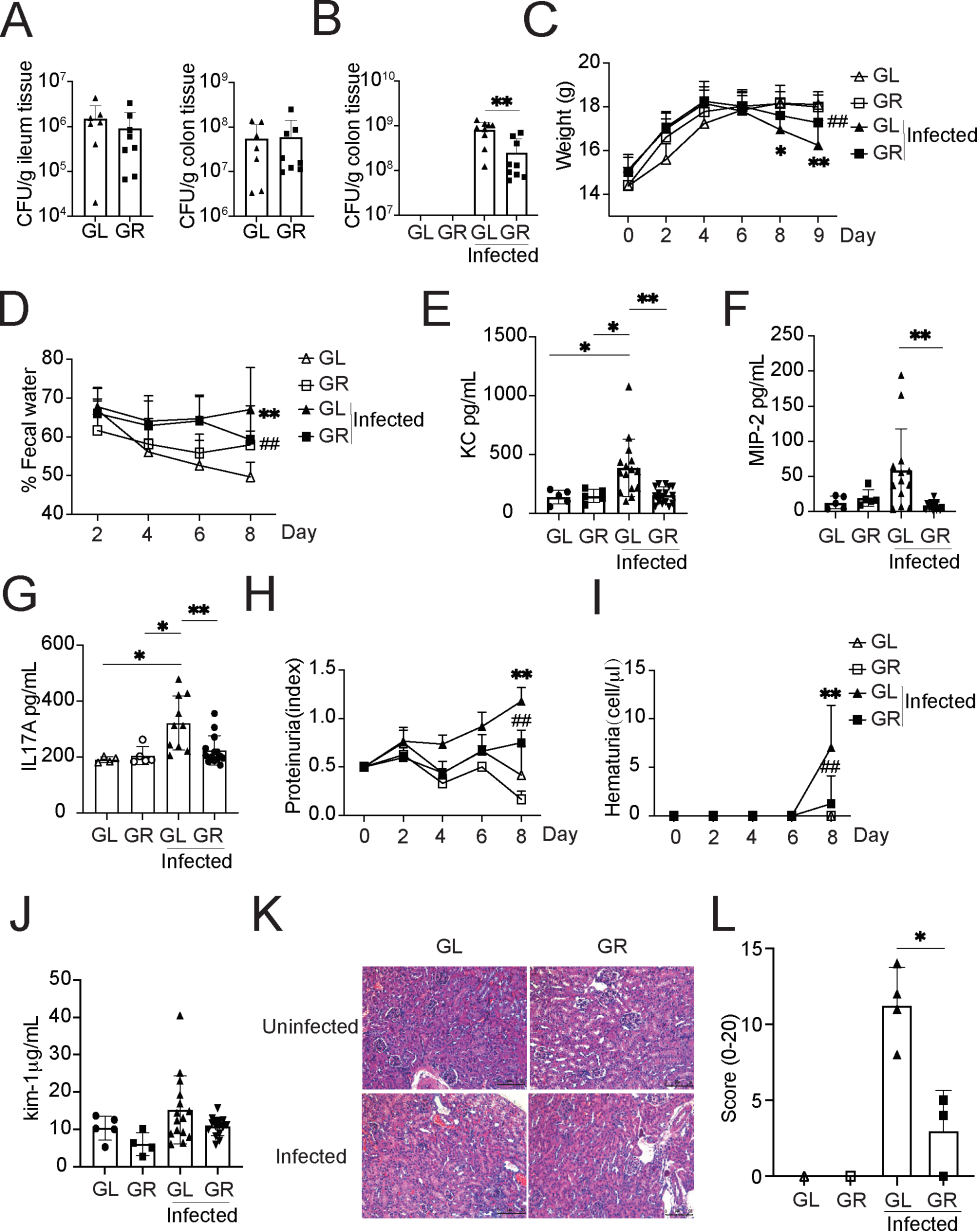
Glutamine (Gln) protects against Stx-producing *C. rodentium* (CR) infection *in vivo*. (**A**) Adherence capacity of CR in the ileum, cecum, and colon of mice fed with a Gln-limited diet (GL) or a Gln-rich diet (GR) at 6 h postinfection. Each symbol represents an individual mouse. Statistics were performed using an unpaired *t* test. Data are mean ± standard deviation (SD). (**B**) Adhesion capacity of CR (λStx_2dact_) in the colon of mice fed with GL or GR at 9 d postinfection. Each symbol represents an individual mouse. Statistics were performed using ordinary one-way ANOVA multiple comparisons. Data are mean ± standard deviation (SD). **P < 0.01. (**C**)Body weight of mice during infection. Statistics were performed using two-way ANOVA multiple comparisons. Data are mean ± standard deviation (SD). *P < 0.05, **P < 0.01, ^##^ *P* < 0.01. *GL-infected group compared to GL-uninfected group, ^#^GR-infected group compared to GL-infected group. (**D**)Fecal water content of infected and uninfected mice fed with GL or GR. Statistics were performed using two-way ANOVA multiple comparisons. Each symbol represents an individual mouse. Data are mean ± standard deviation (SD). **P < 0.01, ^##^ *P* < 0.01. *GL-infected group compared to GL-uninfected group, ^#^GR-infected group compared to GL-infected group. (**E through G**) Stx-mediated systemic induction of proinflammatory cytokines during murine infection with CR (λStx_2dact_). The concentrations of KC, MIP-2, and IL-17 in serum at 9 d were determined by ELISA kits. Each symbol represents an individual mouse. Statistics were performed using ordinary one-way ANOVA multiple comparisons. Data are mean ± standard deviation (SD). *P < 0.05, **P < 0.01, ***P < 0.001. (**H and I**) Proteinuria in infected mice or uninfected mice. Each symbol represents an individual mouse. The range of protein measured was 0–500 mg/dL, with 0 indicating undetectable protein, 0.5 indicating trace, 1.0 indicating ~30 mg/dL, 2.0 indicating ~100 mg/dL, and 3.0 indicating ~500 mg/dL. Hematuria in infected mice or uninfected mice. The range of hematuria was 0–250 erythrocytes/μL, with 0 indicating no hematuria, 5.0 indicating trace, 10.0 indicating ~10 erythrocytes/μL, 25.0 indicating ~25 erythrocytes/μL, and 80.0 indicating ~80 erythrocytes/μL. Statistics were performed using two-way ANOVA multiple comparisons. Data are mean ± standard deviation (SD). **P < 0.01, ^##^ *P* < 0.01. *GL-infected group compared to GL-uninfected group, ^#^GR-infected group compared to GL-infected group. (**J**) The concentrations of kidney injury marker 1 (Kim-1) in urine on day 6 were determined by ELISA kits. Statistics were performed using ordinary one-way ANOVA multiple comparisons. Data are mean ± standard deviation (SD). (**K**) Micrographs (×200) of H&E-stained kidney sections from mice after infection at 9 d. *n* = 3. (**L**) Histological score of the colon 9 d after CR (λStx_2dact_) infection (*n* = 3–4). Each symbol represents an individual mouse. Statistics were performed using ordinary one-way ANOVA multiple comparisons. Data are mean ± standard deviation (SD). *P < 0.05.

Apart from inhibiting bacterial T3SS, could Gln protect against bacterial infection by boosting host defense? To address this question, Stx-producing CR was used in our study to investigate whether bacterial infection, especially Stx-associated damage, could be attenuated by Gln administration *in vivo.* Released Stx often results in severe tissue damage and is the main cause of mortality in EHEC infection. However, EHEC does not cause long-term infection or pathological symptoms significantly in mice due to its inherent resistance to infection. As PchA-Ler regulation was naturally omitted in CR and its T3SS was found undamaged in the presence of Gln as shown above, a CR strain (DBS770) lysogenized by an Stx_2dact_-producing phage, which shares an infection strategy and *stx* gene with EHEC, provides a physiologically relevant model for this study in conventional mice (37). Mice were provided with water with Gln (GR) or without Gln (GL) and 1 week later orally infected with CR. To further understand the effect of Gln on bacterial adhesion *in vivo*, the numbers of intestinal attached CR were counted at 6 h postinfection. In contrast to the results of EHEC O157 colonization *in vivo* (Fig. 4B through D), Gln administration did not reduce bacterial loads of CR in infected mice at 6 h postinfection (*n* = 7–8) (Fig. 7A), which was unsurprising as our *in vitro* experiment had suggested that Gln supplementation did not attenuate T3SS in CR (Fig. 3D; Fig. S4). However, the numbers of bacteria attached to colonic tissue were found reduced in GR mice at a longer time point (*n* = 9) (9 d postinfection) (Fig. 7B). At the end of the experiment, it was also obvious that infected GL mice weighed less than mice in other groups (Fig. 7C). It was observed that multiple Stx-relevant pathological hallmarks had been improved by Gln administration. The stools from infected mice were relatively wet (water content ~65%) on days 2, 4, and 6 postinfection, but by day 8 postinfection, the water content in the stools from GR mice dropped to ~60%, while the water content in the stools from GL mice remained at ~65% (Fig. 7D). The Stx-mediated immune response was evaluated by the production of host chemokines, such as CXCL1/KC, CXCL2/MIP-2, and IL-17A. Gln administration significantly reduced the generation of these chemoattractants in infected mice (Fig. 7E through G). As kidney injury caused by Stx was often reflected by proteinuria or hematuria, these two clinical predictors were also measured in our study. It is obvious that GL mice soon suffered from proteinuria and hematuria after infection. However, GR mice demonstrated healthy conditions after infection, which was nearly indistinguishable from uninfected mice (Fig. 7H and I). Also, kidney injury marker 1 (Kim-1) was found less in infected GR mice relative to that found in infected GL mice (Fig. 7J). To examine organ/tissue damage caused by bacterial infection, kidney/colon samples were collected for pathology analysis on day 9 postinfection. Histologically, the kidneys of GL mice infected with CR DBS770 showed proximal tubule injury that was alleviated in the kidneys of infected GR mice (Fig. 7K). Infected GL mice displayed increased colonic inflammation, submucosal edema, goblet cell depletion, epithelial hyperplasia, and damage (Fig. S6A) and much higher colonic pathology scores relative to infected GR mice (Fig. 7L). H&E (hematoxylin-eosin) sections from liver/spleen samples further support that Gln administration attenuated inflammation and damage caused by CR DBS770 *in vivo* (Fig. S6B).

### Gln reduces mortality in a Δ*Tlr4* mouse model for CR infection

**Fig 8:**
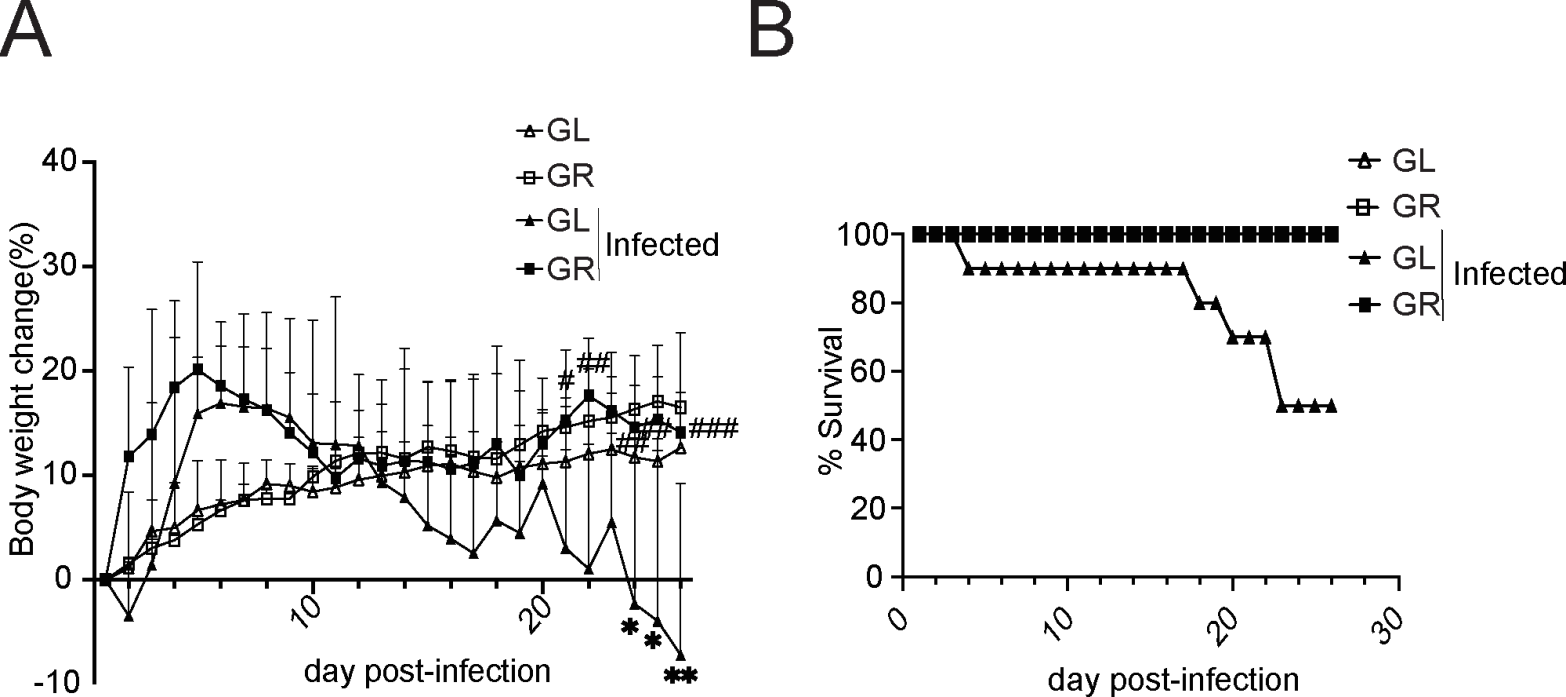
Glutamine (Gln) reduces mortality in a *Tlr4* mouse model for *C. rodentium* (CR) infection. (**A**)Body weight of mice during infection, expressed as percent change from day 0. Statistics were performed using two-way ANOVA multiple comparisons. Data are mean ± standard deviation (SD). *P < 0.05, **P < 0.01, ^#^ *P* < 0.05, ^##^ *P* < 0.01, ^###^ *P* < 0.001. *GL-infected group compared to GR-uninfected group, ^#^GR-infected group compared to GL-infected group. (**B**)Percent survival of groups of *Tlr4* deletion mice that were fed with a Gln-limited diet (GL) or a Gln-rich diet (GR) after infected with CR (DBS100n).

Toll-like receptor 4 (TLR4)-mediated immunomodulation was proved important for antibacterial infection, which recognizes pathogen-associated molecular patterns (PAMPs), such as lipopolysaccharide (LPS) from Gram-negative bacteria, and subsequently activates both innate and adaptive immune cells in the host (38, 39). Gln was previously shown to attenuate host inflammation by modulating the TLR4 signaling pathway, and therefore, in this study, we were keen to know whether TLR4 played a key role in Gln protection for CR-infected mice. To validate this hypothesis, a lethal infection model was carried out in mice with *Tlr4* deletion, which is highly susceptible to CR. CR strain DBS100n was used in this experiment in order to remove the effect caused by Stx. In this experiment, before infection, mice were treated with different diets (GL or GR) for a week. One group of GL mice was kept as uninfected controls, and other mice were orally infected with CR (1 × 10^6^ CFU). Although GL mice rapidly became ill from the infection, showing signs of disease, GR mice remained healthy after infection. In our study, it was clearly shown that GR mice (*n* = 9; originally, there were 10 mice in this group, but one died from suffocating accidentally on day 20 postinfection) maintained stable body weights after infection, while GL mice (*n* = 10) kept losing body weights since day 5 postinfection (Fig. 8A). And a 100% survival rate (*n* = 9) was observed in infected GR mice, and only a 50% survival rate (*n* = 10) was observed in infected GL mice in our experiment (Fig. 8B). Thus, our evidence suggested that dietary Gln supplementation could protect against CR infection in TLR4-deficient mice.

## DISCUSSION

The gastrointestinal environment is complicated and profoundly influenced by the gut microbiome, host genetics, diet, and intestinal diseases (40). EHEC has evolved a delicate regulatory response to multiple cues to finely tune its gene expression for colonizing the densely populated colon (41). Emerging evidence revealed that niche signals could be crucial for bacterial virulence expression and its adaptation to the host environment (42). Also, many studies have proved that bacterial lifestyle was profoundly impacted by metabolites of the host/microbiota (13, 40, 43). For example, several studies indicated that quorum sensing signals could activate the expression of EHEC LEE genes *via* QseC/E and that endocannabinoid 2-arachidonoylglycerol (2-AG) could inhibit T3SS activation by antagonizing QseC (12, 44). Recently, another study revealed that long-chain fatty acids affected intestinal colonization of EHEC by repressing LEE expression *via* FadR (45). Moreover, arginine directly activates the expression of genes encoding T3SS through the arginine sensor ArgR (24), while EA, a vital component of bacterial and mammalian cell membranes, can enhance EHEC infection to host cells (43). These studies demonstrated the complexity of host-pathogen interactions and also provided useful information for anti-infection solutions.

As the most abundant amino acid in the human body, Gln was found to be 10~100-fold more than any other amino acids in human organs/tissues, and it was also the main source of energy for cells in the lumen of the gut (14, 15). Although the gut was considered the major site of Gln absorption/consumption, many previous research studies have indicated that the concentration of Gln was much lower in intestinal tissues compared to the concentration in other tissues due to high levels of glutaminase activity. Our findings above indicated that Gln limitation could act as a stimulus for EHEC colonization considering the concentration of Gln in colon epithelial cells/mucosa. In this study, we found that Gln supplementation reduced the expression of EHEC T3SS *via* the perturbation of bacterial central metabolism. Gln availability could shift the phosphorylation of NtrC in EHEC, and phosphorylated NtrC was proved to activate *ler* transcription through the σ^S^-PchA pathway in our study. At the same time, our evidence suggested that exogenous addition of α-KG significantly activated EHEC T3SS possibly in the same way (data not shown). Furthermore, Gln does not inhibit EHEC growth (Fig. S5A), while it could repress T3SS *via* bacterial central metabolism. Therefore, it was speculated that Gln-resistant strains should be rarely found, as the dysfunctional mutation of central metabolism-related genes might cause negative impact on bacterial growth or fitness.

Traditional antibiotic treatment for bacterial infections might damage the host microbiota and also raise problems of antimicrobial-resistant bacterial pathogens. The antivirulence strategy was considered a promising solution for this dilemma. Moreover, it was still lacking therapeutic agents for infections caused by EHEC clinically, as antimicrobial treatment was not recommended due to toxin release from lysed bacteria. In the last 20 yr, many compounds have been tested as antivirulence agents for their roles of anti-infection. Some of them demonstrated positive results for drug development, and the underlying mechanisms of these compounds were helpful for novel drug designs. For example, Rasko et al*.* identified a small molecule (LED209) inhibiting bacterial virulence by targeting the quorum sensing system (46). Also, many studies have been carried out with salicylidene acylhydrazide compounds, which could repress bacterial virulence *via* multiple targets (47). Nowadays, Gln was routinely used as a nutrition supplement for pre/post-operative patients. In our study, the impact of Gln was tested both *in vitro* and *in vivo* for EHEC infection. Encouragingly, Gln demonstrated its own advantages over other antivirulence agents for EHEC infection, as it not only represses bacterial virulence as an antivirulence compound but also enhances the host defense system to minimize the impact caused by infection. Furthermore, in our study, Gln protection against CR infection in ΔTlr4 mice suggested that Gln could impact host defense not only *via* the TLR4 pathway but also *via* other alternates.

Bacterial growth advantage can affect the efficacy of traditional antimicrobial agents due to fitness. It was observed in our study that the bacterial amount was not changed at the stationary phase, while the bacterial growth rate was accelerated at the logarithmic phase by Gln addition under *in vitro* culture conditions (Fig. S5A). However, not like antibiotics that kill the bacterium or inhibit its growth, Gln inhibits the expression of bacterial virulence, which is critical for bacterial infection. Therefore, impaired bacterial pathogens were either no longer able to colonize or diminished by the immune system due to the anti-infection duality of Gln, which was reflected by the reduced bacterial number *in vivo*. It was supported by our evidence that the amount of bacterial pathogens was decreased *in vivo* upon Gln administration.

Previously, Gln supplementation has been extensively studied earlier, and it was confirmed that only 50–80% of dietary Gln was absorbed by the intestine and that millimolar levels of Gln concentration (>2.5 mmol/L) could be achieved in the gut lumen by oral/enteral supplementation (48 – 50). Consequently, many studies were carried out to increase Gln concentration in the intestine using a method of dietary supplementation (51 – 53). In this study, we also adapted a similar methodology in our *in vivo* experimental model. As the aim of this experiment was to investigate the impact of additional Gln on bacterial infection *in vivo*, we did not measure the exact Gln concentration and assumed that Gln concentration would be increased in the intestinal tract by oral/enteral supplementation. However, high levels of Gln concentration might be sustainable only for the short term in the gastrointestinal tract due to degradation and absorption. Therefore, more hydrolytically stable molecules, such as L-alanyl-L-glutamine (DIP), could be evaluated as an alternate for free L-glutamine. Also, sustained-release capsules might be a useful delivery tool to maintain Gln concentration *in vivo* to combat bacterial infection.

Noteworthy, we also revealed other amino acids that might have similar effects as Gln on EHEC infection. These amino acids include, but might not be limited to, L-glutamate, L-asparagine, and L-aspartate (data not shown). Although it still requires more testing and further validation clinically, our study here demonstrated that Gln could protect against EHEC infection by limiting bacterial T3SS and modulating host defense without jeopardizing commensal flora and therefore hinted at repurposing Gln as an anti-infection agent.

## Data Availability

The RNA-seq and 16S-seq raw fastq files are deposited at the NCBI SRA database. RNA-seq data accession library IDs: SRR23625956 ; SRR23625957; SRR23625958; SRR23625959; SRR23625960; SRR23625961; SRR23625962; SRR23625963; SRR23625964; SRR23625965. 16S-seq data accession library IDs: SRR23617005; SRR23617006; SRR23617007; SRR23617008; SRR23617009; SRR23617010; SRR23617011; SRR23617012; SRR23617013; SRR23617014.

## SUPPLEMENTAL MATERIAL

Fig. S1 spectrum.00975-23-s0001.tif Gln inhibits EHEC T3SS in a dose-dependent manner.

Fig. S2 spectrum.00975-23-s0002.tif Gln repressed EHEC T3SS via modulating LEE expression.

Fig. S3 spectrum.00975-23-s0003.tif Transcriptional regulation of ler is mediated by phosphorylated NtrC via σS and PchA.

Fig. S4 spectrum.00975-23-s0004.tif Glutamine does not repress T3SS in C. rodentium.

Fig. S5 spectrum.00975-23-s0005.tif Glutamine supplementation decreases EHEC colonization on cell.

Fig. S6 spectrum.00975-23-s0006.tif Glutamine protects against Stx-producing C. rodentium infection in vivo.

Supplemental material legends spectrum.00975-23-s0007.docx Legends of Fig. S1 to S6.

Table S1 spectrum.00975-23-s0008.pdf Bacterial strains, cell lines, and plasmids used in this study.

Table S2 spectrum.00975-23-s0009.pdf Oligonucleotides used in this study.

Table S3 spectrum.00975-23-s0010.xlsx Differentially regulated genes.

ASM does not own the copyrights to Supplemental Material that may be linked to, or accessed through, an article. The authors have granted ASM a non-exclusive, world-wide license to publish the Supplemental Material files. Please contact the corresponding author directly for reuse.
