# Correction for Fang et al., “L-glutamine protects against enterohemorrhagic *Escherichia coli* infection by inhibiting bacterial virulence and enhancing host defense concurrently”

**DOI:** 10.1128/spectrum.02369-25

**Published:** 2025-08-22

**Authors:** Fang Fang, Yunxin Xue, Xuefang Xu, Dingli Fang, Weijia Liu, Ying Zhong, Jinping Han, Yunhe Li, Qian Tao, Rong Lu, Cong Ma, Arvind Kumar, Dai Wang

## AUTHOR CORRECTION

Volume 11, no. 6, e00975-23, 2023, https://doi.org/10.1128/spectrum.00975-23. Page 1, byline: Dai Wang should have an additional affiliation: “Shenzhen Research Institute of Xiamen University, Shenzhen, Guangdong, China.”

