# Supplementary figures and images for: L-glutamine protects against enterohemorrhagic Escherichia coli infection by inhibiting bacterial virulence and enhancing host defense concurrently
Source: Microbiol Spectr. 2023 Oct 10;11(6):e00975-23. doi: 10.1128/spectrum.00975-23 (PMC10714755; doi:10.1128/spectrum.00975-23)

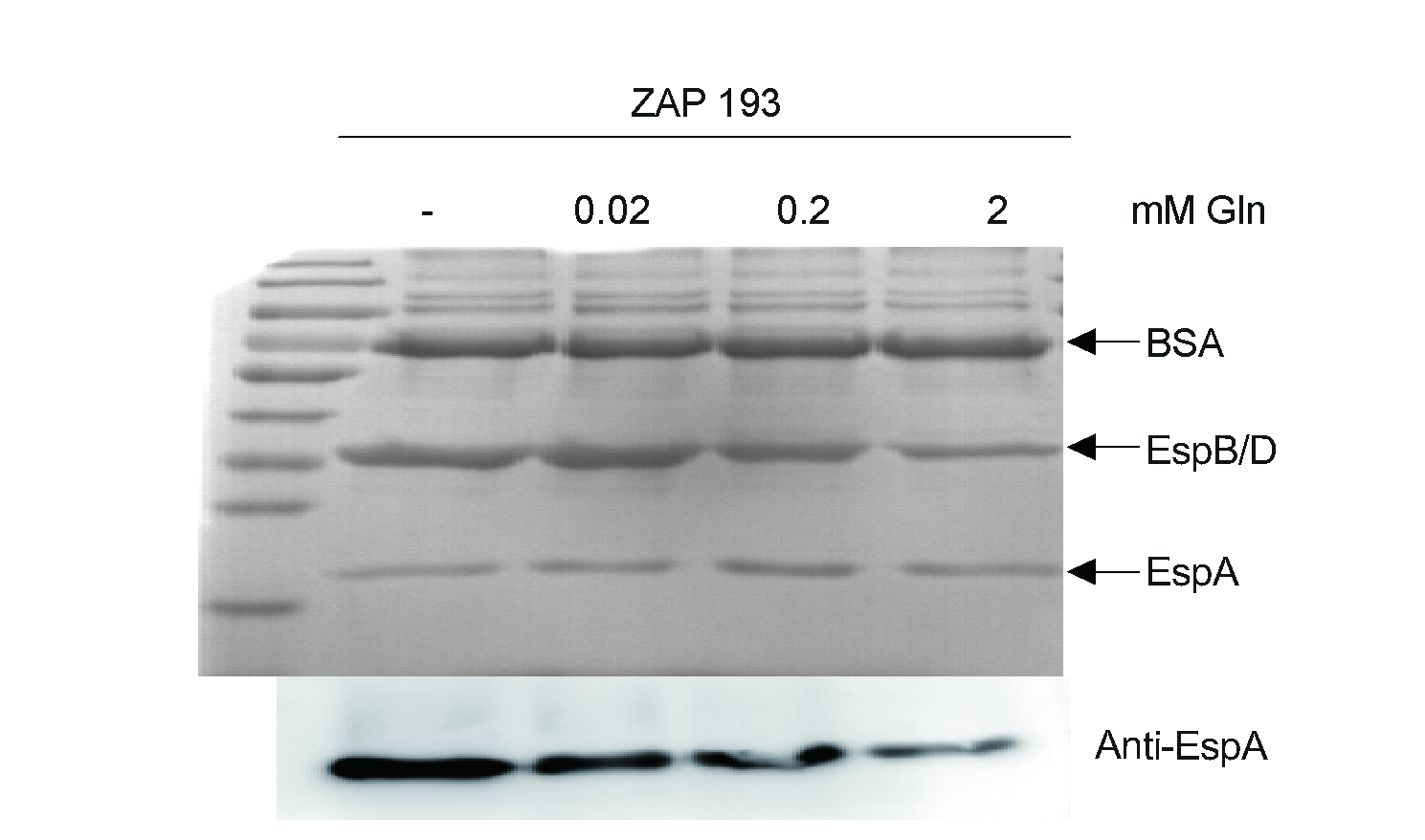

Supplement: Fig. S1 — Gln inhibits EHEC T3SS in a dose-dependent manner. [file spectrum.00975-23-s0001.tif]

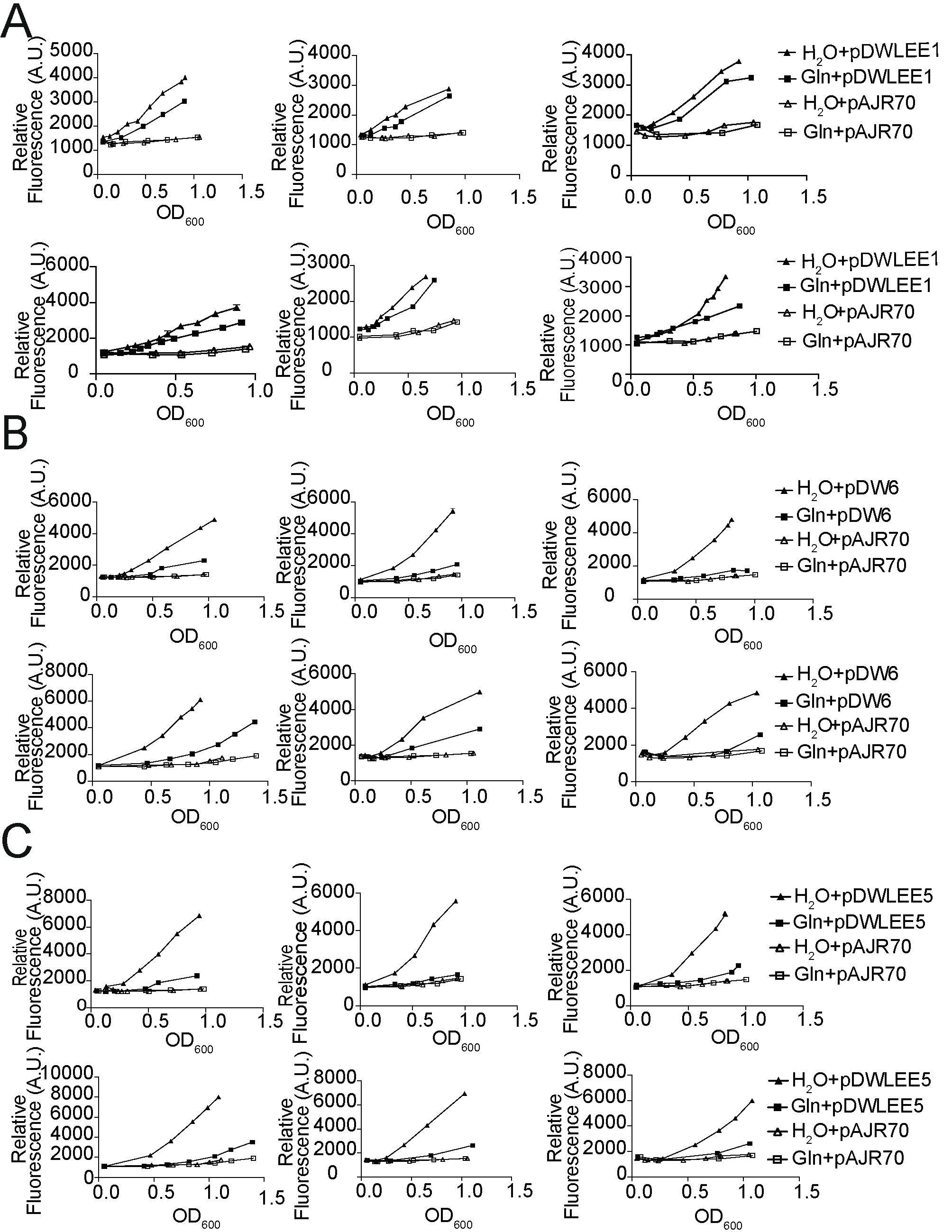

Supplement: Fig. S2 — Gln repressed EHEC T3SS via modulating LEE expression. [file spectrum.00975-23-s0002.tif]

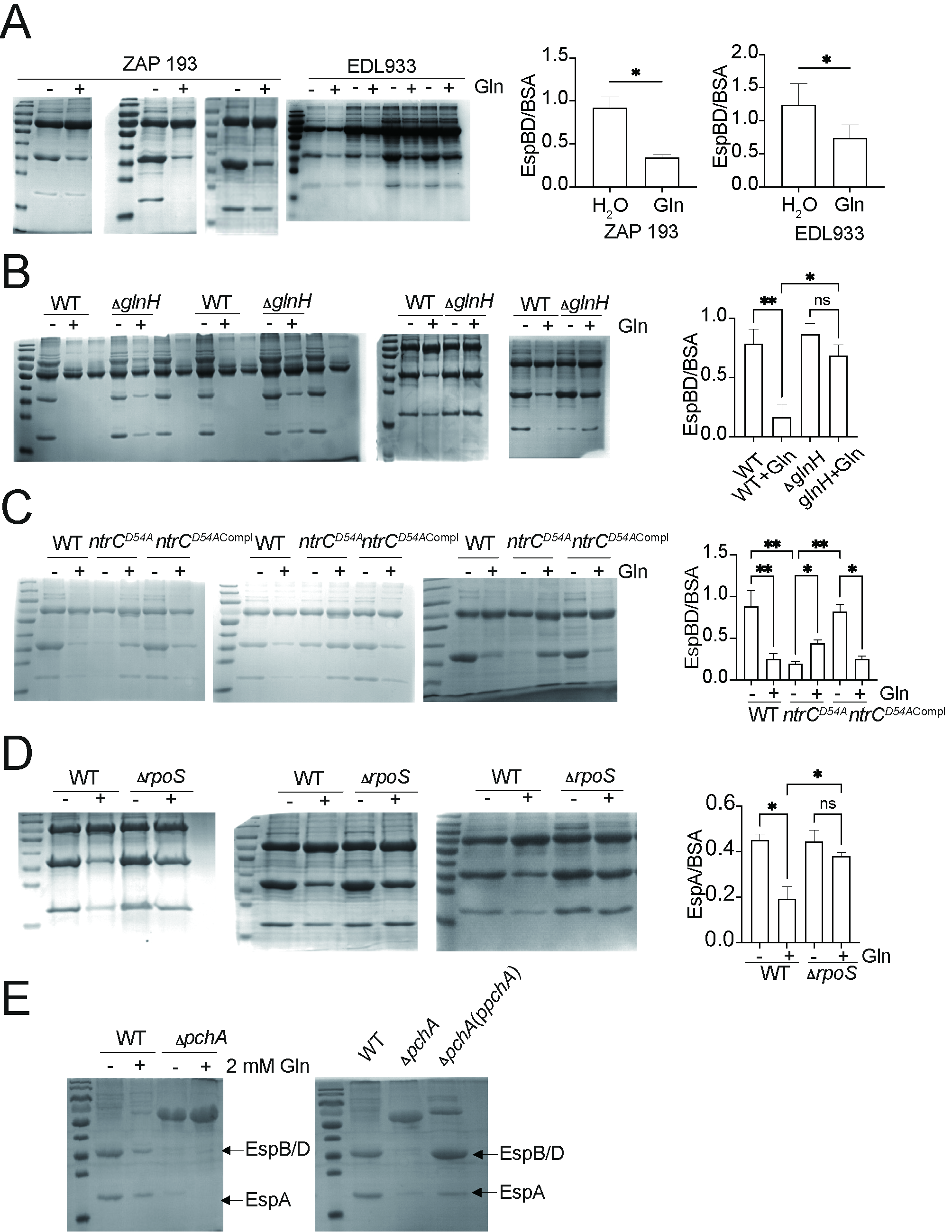

Supplement: Fig. S3 — Transcriptional regulation of ler is mediated by phosphorylated NtrC via σS and PchA. [file spectrum.00975-23-s0003.tif]

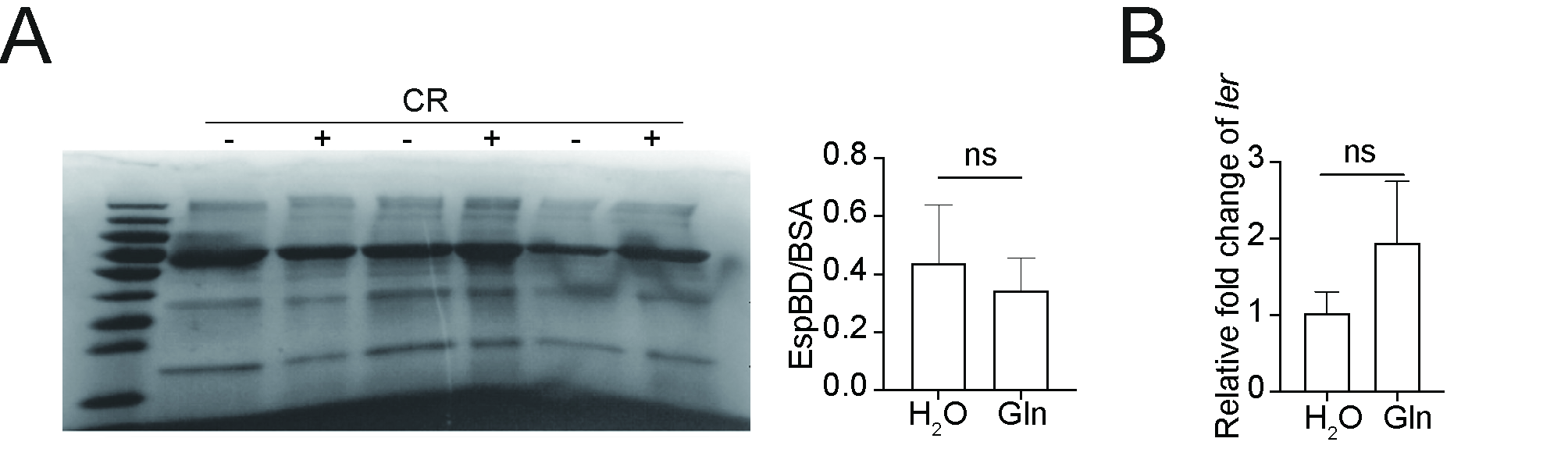

Supplement: Fig. S4 — Glutamine does not repress T3SS in C. rodentium. [file spectrum.00975-23-s0004.tif]

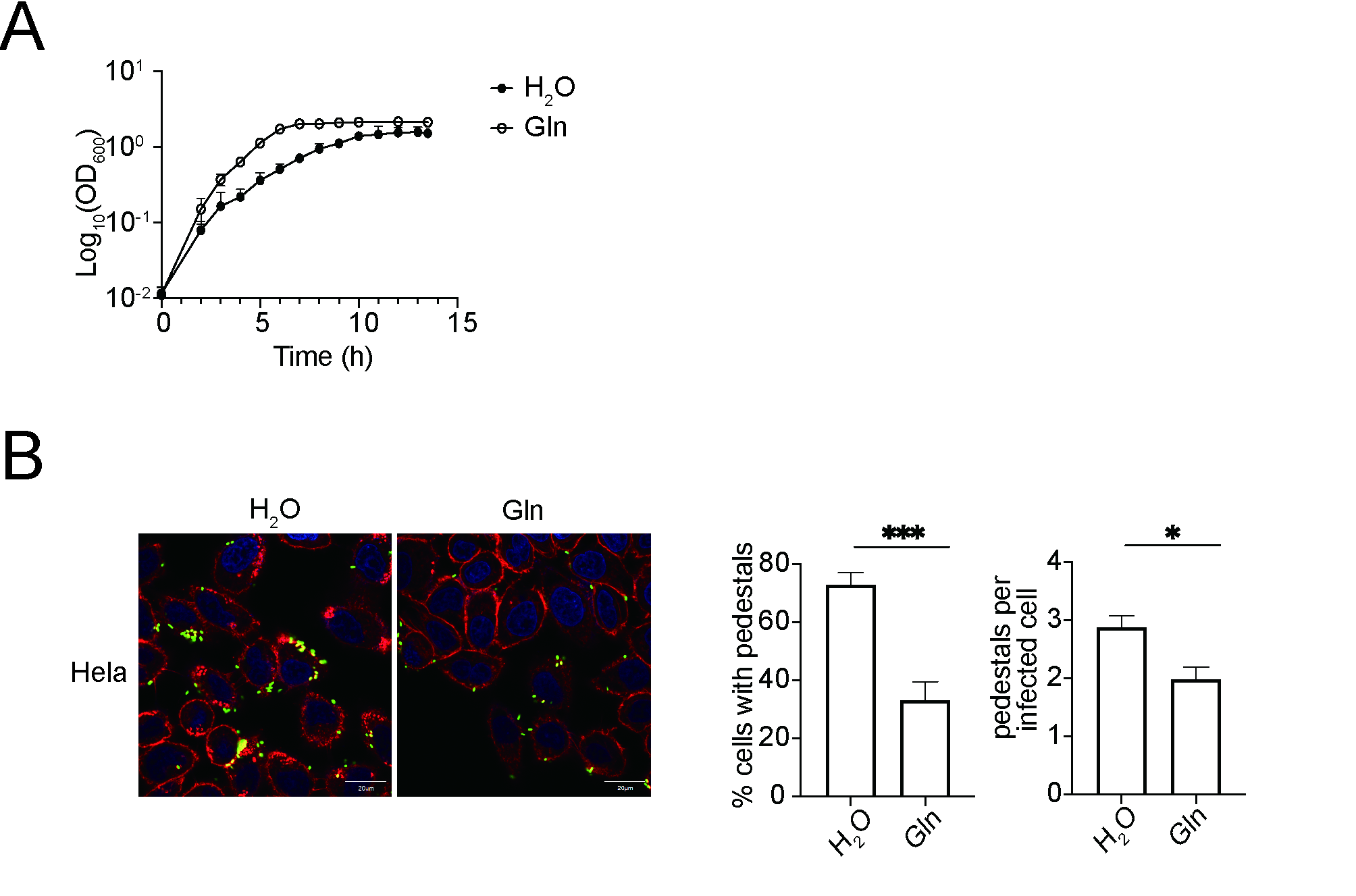

Supplement: Fig. S5 — Glutamine supplementation decreases EHEC colonization on cell. [file spectrum.00975-23-s0005.tif]

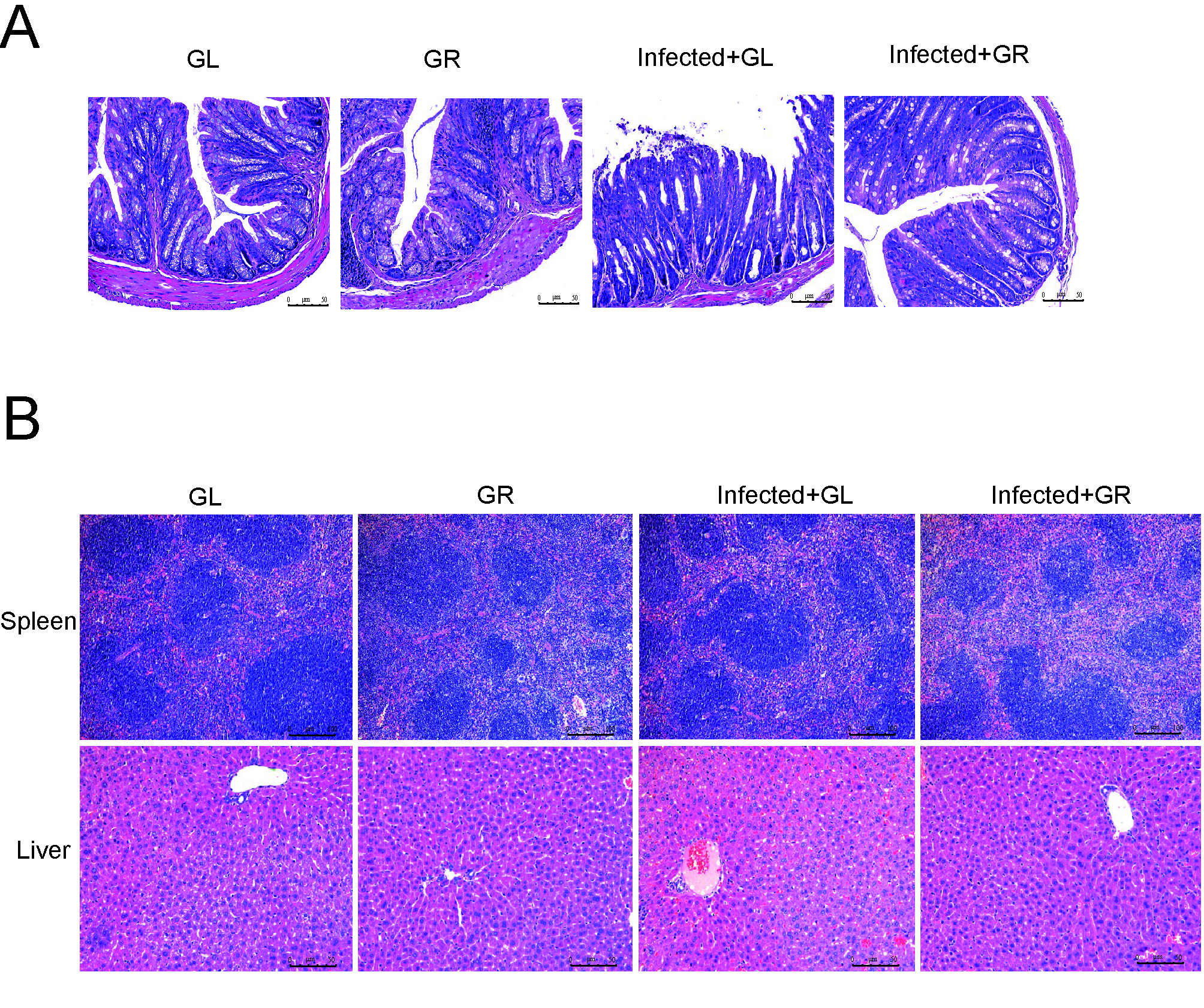

Supplement: Fig. S6 — Glutamine protects against Stx-producing C. rodentium infection in vivo. [file spectrum.00975-23-s0006.tif]
