# Supplementary material for: L-glutamine protects against enterohemorrhagic Escherichia coli infection by inhibiting bacterial virulence and enhancing host defense concurrently
Source: Microbiol Spectr. 2023 Oct 10;11(6):e00975-23. doi: 10.1128/spectrum.00975-23 (PMC10714755; doi:10.1128/spectrum.00975-23)
Supplement: Supplemental material legends — Legends of Fig. S1 to S6. [file spectrum.00975-23-s0007.docx]

**Fig S1 Gln inhibits EHEC T3SS in a dose-dependent manner.**

T3 secreted proteins of EHEC were visualized using Coomassie blue staining/EspA immuno-blotting following cultured with different concentrations of Gln.

**Fig S2** Gln repressed EHEC T3SS *via* modulating LEE expression

(A)Expression levels of Ler (pDWLEE1), (B)SepL (pDW6), (C)Tir (pDWLEE5) and empty control (pAJR70) were monitored with GFP fusion plasmids in EHEC strain ZAP193 when culturing with or without 2 mM Gln.

**Fig S3 Transcriptional regulation of *ler* is mediated by phosphorylated NtrC via σS and PchA**

(A) T3 secreted proteins of EHEC were visualized using Coomassie blue staining. Protein levels were semiquantified using ImageJ. The relative intensity in shown as the ratio of the signal intensity of EspB/D to BSA (loading control) in ZAP 193or EDL933 following cultured with or without 2 mM Gln supplementation. Statistics were performed using unpaired t test. Data are mean±standard deviation (SD), n=3-4, *P < 0.05.

(B) T3 secreted proteins of EHEC were visualized using Coomassie blue staining. The relative intensity in shown as the ratio of the signal intensity of EspB/D to BSA (loading control) in WT and ∆*glnH* following cultured with or without 2 mM Gln supplementation. Statistics were performed using ordinary one-way ANOVA multiple comparisons. Data are mean±standard deviation (SD), n=4, *P < 0.05.

(C) T3 secreted proteins of EHEC were visualized using Coomassie blue staining. The relative intensity in shown as the ratio of the signal intensity of EspB/D to BSA (loading control) in WT, *ntrC^D54A^* and *ntrC^D54A::ntrC^* following cultured with or without 2 mM Gln supplementation. Statistics were performed using ordinary one-way ANOVA multiple comparisons. Data are mean±standard deviation (SD), n=5, *P < 0.05.

(D) The relative intensity in shown as the ratio of the signal intensity of EspA to BSA (loading control) in WT, and ∆*rpoS* following cultured with or without 2 mM Gln supplementation. Statistics were performed using ordinary one-way ANOVA multiple comparisons. Data are mean±standard deviation (SD), n=3, P < 0.05,**P < 0.01.

(E) The secreted protein profile of EHEC wild type and ∆*pchA* strains were visualized using Coomassie blue staining following cultured with or without 2 mM Gln supplementation (Left)*.* T3 secreted proteins of EHEC wild type, ∆*pchA* and ∆*pchA^::ppchA^* strains were visualized using Coomassie blue staining (Right).

**Fig S4 Glutamine does not repress T3SS in *C. rodentium***

(A) T3 secreted proteins of EHEC were visualized using Coomassie blue staining. The relative intensity in shown as the ratio of the signal intensity of EspBD to BSA (loading control) in CR following cultured with or without 2 mM Gln supplementation. Statistics were performed using ordinary one-way ANOVA multiple comparisons. Data are mean±standard deviation (SD), n=3, ns：no significant. (B) Transcript levels of *ler* were evaluated by quantitative real-time PCR in CR (n=3). Statistics were performed using unpaired t test. Data are mean±standard deviation (SD),n=3, ns：no significant.

**Fig S5 Glutamine supplementation decreases EHEC colonization on cell.**

(A) *In vitro* growth curve of EHEC cultured in absence or presence of 2 mM Gln. Data are mean±standard deviation (SD).

(B) AE lesion formation on Hela cells by EHEC in the absence or presence of 2 mM Gln. EHEC forms AE lesions on epithelial cells, whose hallmark is actin accumulation underneath the bacterial cell forming a pedestal-like structure. EHEC were probed with O157 antibody and Alexaflour 488 conjugated secondary antibody (green). Cells were stained with FITC-phalloidin (Actin, colored in Red) and DAPI (DNA, colored in Blue) and observed with fluorescence microscopy (100×), Scale bars, 20 μm. Percentage of cells infected with EHEC pedestals and quantification of EHEC pedestals per infected cell. Statistics were performed using unpaired t test. Data are mean±standard deviation (SD), *P < 0.05,***P < 0.0001.

**Fig S6 Glutamine protects against Stx-producing *C. rodentium* infection *in vivo*.**

1. Representation (200×) of colon 9 d after C.*rodentium* (λStx_2dact_) infection (n=3-4).

(B) Representation of Spleen (100×) and Liver (200×) 9 d after C.*rodentium* (λStx_2dact_) infection (n=3-4).
