# Supplementary material for: L-glutamine protects against enterohemorrhagic Escherichia coli infection by inhibiting bacterial virulence and enhancing host defense concurrently
Source: Microbiol Spectr. 2023 Oct 10;11(6):e00975-23. doi: 10.1128/spectrum.00975-23 (PMC10714755; doi:10.1128/spectrum.00975-23)
Supplement: Table S1 — Bacterial strains, cell lines, and plasmids used in this study. [file spectrum.00975-23-s0008.pdf]

Table S1 **Bacterial strains, cell lines and plasmids used in this study.**

Strains	Description	Source or reference
DBS100n	Derived from *Citrobacter rodentium* strain DBS100, Nal^R	Lab stock
DBS770	*Citrobacter rodentium* (λstx_{2dact}), Cm^R	Gift from Prof. JM Leong
ZAP193	*E. coli* O157:H7, Stx negative, Nal^R, NCTC 12900	(1)
EDL933	*E. coli* O157:H7	(2)
Δ*glnH*	ZAP193 Δ*glnH*	This work
Δ*pchA*	ZAP193 Δ*pchA*	This work
$ntrC^{D54A}$	ZAP193 $ntrC^{D54A}$	This work
$ntrC^{D54A\text{Compl}}$	ZAP193 $ntrC^{D54A}$ complemented with wild type *ntrC* gene on chromosome	This work
Δ*pchA*	ZAP193 Δ*pchA*	This work
Cell lines		
Hela	Human cervical carcinoma cell line	Lab stock
Caco-2	Human colonic adenocarcinoma cell line	Lab stock
Plasmids		
pWSK29	Expression vector, Amp^R	Lab stock
p*pchA*	pWSK29 carrying *pchA,* under the control of the IPTG-inducible trc promoter	This work
pCas	Kan^R, repA101 (temperature- sensitive replicon), lacIq-Ptrc fragment, sg-RNA-pMBI, λ-Red recombinase gene (exo bet gam paraB)	(3)
pTargetF	aadA ($Spec^R$), pMB1, pj23119 (synthetic promoter) N20-sgRNA	(3)
pTargetF-sgRNA-*glnH*	$Spec^R$, sgRNA with an N20 sequence for targeting the partial *glnH* fragment from wildtype, with the homologous fragment of *glnH*	This work
pTargetF-sgRNA-*pchA*	$Spec^R$, sgRNA with an N20 sequence for targeting the partial *pchA* fragment from wildtype, with the homologous fragment of *pchA*	This work
pTargetF-sgRNA-$ntrC^{D54A}$	$Spec^R$, sgRNA with an N20 sequence for targeting the partial $ntrC^{D54A}$ fragment from wildtype, with the homologous fragment of $ntrC^{D54A}$	This work
pTargetF-sgRNA-$ntrC^{A54D}$	$Spec^R$, sgRNA with an N20 sequence for targeting the mutated *ntrC* sequence from $ntrC^{D54A}$, with the homologous fragment of *ntrC* (wild type sequence)	This work

pACYC184	Low copy number cloning vector	New England Biolabs
pAJR70	pACYC184 digested with *BamH* I; *gfp* gene cloned *BamH* I/ *Bgl* II	(4)
pDW6	pAJR70 digested with *BamH* I/ *Kpn* I; *sepL* with its own promoter amplified from ZAP193, cloned in frame 5' to *gfp*	(4)
pDWLEE1	pAJR70 digested with *BamH* I/ *Kpn* I; *ler* with its own promoter amplified from ZAP193, cloned in frame 5' to *gfp*	(4)
pDWLEE5	pAJR70 digested with *Bgl* II /*Kpn* I; *tir* with its own promoter amplified from ZAP193, cloned in frame 5' to *gfp*	(4)

Cm^R, Chloramphenicol resistance; $Spec^R$, Spectinomycin resistance; Amp^R, Ampicillin resistance; Kan^R, Kanamycin resistance; Nal^R, Nalidixic acid resistance

1. Wang, D., Roe, A. J., McAteer, S., Shipston, M. J., and Gally, D. L. 2008 Hierarchal type III secretion of translocators and effectors from *Escherichia coli* O157:H7 requires the carboxy terminus of SepL that binds to Tir. Mol Microbiol 69: 1499-1512
2. Wells, T. J., Sherlock, O., Rivas, L., Mahajan, A., Beatson, S. A., Torpdahl, M., Webb, R. I., Allsopp, L. P., Gobius, K. S., Gally, D. L., and Schembri, M. A. 2008 EhaA is a novel autotransporter protein of enterohemorrhagic *Escherichia coli* O157:H7 that contributes to adhesion and biofilm formation. Environ Microbiol 10: 589-604
3. Jiang, Y., Chen, B., Duan, C., Sun, B., Yang, J., and Yang, S. 2015 Multigene editing in the *Escherichia coli* genome via the CRISPR-Cas9 system. Appl Environ Microbiol 81: 2506-2514
4. Fernandez-Brando, R. J., Yamaguchi, N., Tahoun, A., McAteer, S. P., Gillespie, T., Wang, D., Argyle, S. A., Palermo, M. S., and Gally, D. L. 2016 Type III Secretion-Dependent Sensitivity of *Escherichia coli* O157 to Specific Ketolides. Antimicrob Agents Chemother 60: 459-470
