# Supplementary material for: L-glutamine protects against enterohemorrhagic Escherichia coli infection by inhibiting bacterial virulence and enhancing host defense concurrently
Source: Microbiol Spectr. 2023 Oct 10;11(6):e00975-23. doi: 10.1128/spectrum.00975-23 (PMC10714755; doi:10.1128/spectrum.00975-23)
Supplement: Table S2 — Oligonucleotides used in this study. [file spectrum.00975-23-s0009.pdf]

Table S2. **Oligonucleotides used in this study**.

| Oligonucleotides used in this study | | |
|---|---|---|
| Name | Used to construct plasmid | Sequence |
| pTargetF-sgRNA-3’ | pTargetF-sgRNA | CTCAAAAAAAGCACCGACTCGG |
| *glnH*-sgRNA-5’ | pTargetF-sgRNA-*glnH* | GGACTAGTGTTIGCTTTCGCGTAATCAAGTTTTAGAG<br>CTAGAAATAGCH |
| *glnH*-up-5’ | pTargetF-sgRNA-*glnH* | CGAGTCGGTGCTTTTTTTGAGGTGCAGAATTTTAGTA<br>CCCG |
| *glnH*-up-3’ | pTargetF-sgRNA-*glnH* | TTGTAAGTTCCGTTCTCGCGCAGTGCAGCCAGTGAA<br>AC |
| *glnH*-down-5’ | pTargetF-sgRNA-*glnH* | GTTTCACTGGCTGCACTGCGCGAGAACGGAACTTAC<br>AA |
| *glnH*-down-3’ | pTargetF-sgRNA-*glnH* | AACTGCAGGATCATGATGGTGACCAC |
| *pchA*-sgRNA-5’ | pTargetF-sgRNA-*pchA* | GGACTAGTTCAGCGTTTAAGGCATTGAAGTTTTAGA<br>GCTAGAAATAGC |
| *pchA*-up-5’ | pTargetF-sgRNA-*pchA* | CGAGTCGGTGCTTTTTTTGAGGCGATCCCTGAAGAT<br>ATATTC |
| *pchA*-up-3’ | pTargetF-sgRNA-*pchA* | CAACAATGAATCATCCCCTTCCGCGGACGATTTGCA<br>CTTATCAT |
| *pchA* -down-5’ | pTargetF-sgRNA-*pchA* | CGGAAGGGGATGATTCATTGTTGA |
| *pchA*-down-3’ | pTargetF-sgRNA-*pchA* | AACTGCAGGACACGCTTTTGTCATTCC |
| *ntrC*$^{D54A}$-sgRNA-5’ | pTargetF-sgRNA-<br>*ntrC*$^{D54A}$ | GGACTAGTTTTATCGCTCTGAATATGGCGTTTTAGA<br>GCTAGAAATAGC |
| *ntrC*$^{D54A}$-up-5’ | pTargetF-sgRNA-<br>*ntrC*$^{D54A}$<br>pTargetF-sgRNA-<br>*ntrC*$^{A54D}$ | CGAGTCGGTGCTTTTTTTGAGCGCGTTTCAACTGACC<br>TTAC |
| *ntrC*$^{D54A}$-up-3’ | pTargetF-sgRNA-<br>*ntrC*$^{D54A}$ | TAACTTACGCGTCAGGGTGCAGACTATCCCTCGTTG<br>CAT |
| *ntrC*$^{D54A}$-down-5’ | pTargetF-sgRNA-<br>*ntrC*$^{D54A}$ | CACCCTGACGCGTAAGTTA |
| *ntrC*$^{D54A}$-down-3’ | pTargetF-sgRNA-<br>*ntrC*$^{D54A}$<br>pTargetF-sgRNA-<br>*ntrC*$^{A54D}$ | AACTGCAGGCTTATCAGGCCTACAAAAC |
| *ntrC*$^{A54D}$-sgRNA-5’ | pTargetF-sgRNA-<br>*ntrC*$^{A54D}$ | GGACTAGTGCTTTCAGCTATACGTATGCGTTTTAGA<br>GCTAGAAATAGC |
| *ntrC*$^{A54D}$-up-3’ | pTargetF-sgRNA-<br>*ntrC*$^{A54D}$ | GGCATACGGATATCTGAAAGC |
| *ntrC*$^{A54D}$-down-5’ | pTargetF-sgRNA-<br>*ntrC*$^{A54D}$ | GCTTTCAGATATCCGTATGCC |
| 341F | 16S rDNA | CCTACGGGNGGCWGCAG |
| 806R | 16S rDNA | GGACTACHVGGGTATCTAAT |

| Oligonucleotides used in RT-PCR | |
|---|---|
| *pchA*-F | GATATTCCCTGGCGACTGGC |
| *pchA*-R | CGGCGGTAAGCCATCTACTC |
| *stx1*-F | GTGGCATTAATACTGAATTGTCATCA |
| *stx1*-R | GCGTAATCCCACGGACTCTTC |
| *stx2a*-F | GATGTTTATGGCGGTTTTATTTGC |
| *stx2a*-R | TGGAAAACTCAATTTTACCTTTAGCA |
| *recA*-F | ATATCGACGCCCAGTTTACG |
| *recA*-R | GTTCCATGGATGTGGAAACC |
| *16S*-F | CTTACGACCAGGGCTACACAC |
| *16S*-R | CGGACTACGACGCACTTTATG |
